# The magnitude of the survival benefit of internal thoracic artery grafting: Absolute risk reduction

**DOI:** 10.1016/j.xjon.2021.11.008

**Published:** 2021-11-20

**Authors:** Takayuki Ohno

**Affiliations:** Cardiovascular Surgery and Intensive Care Unit, Mitsui Memorial Hospital, Tokyo, Japan

**Keywords:** CABG, ITA, treatment effect, all-cause death, myocardial infarction, absolute risk reduction, number needed to treat

## Abstract

The magnitude of the survival benefit of CABG with internal thoracic artery graft increases with time over decades.

**Figure:**
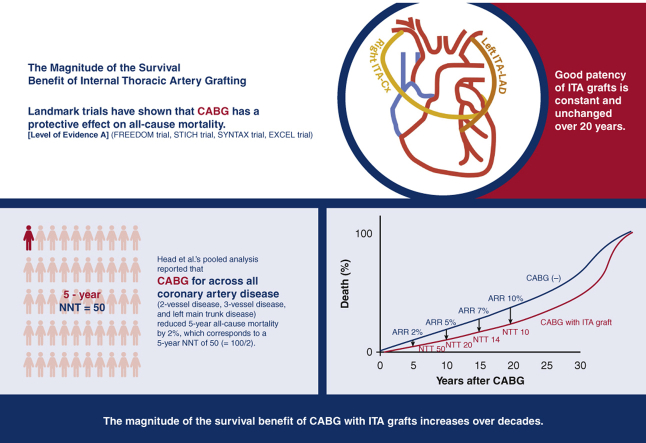

**Figure:**
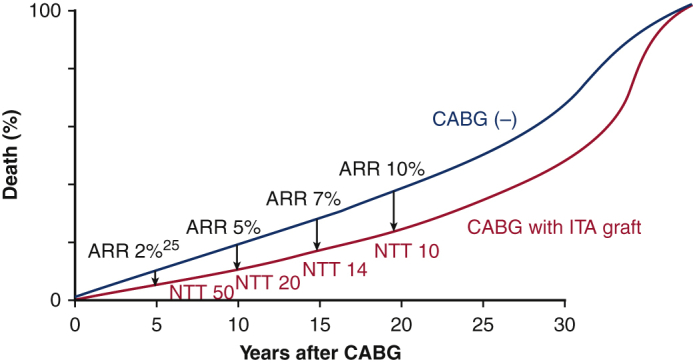
Because death is inevitable for all humans, the survival benefits of CABG will eventually disappear. Although this is a conceptual survival curve, the survival benefit of ITA grafting increases the benefit in terms of survival over decades. The expected absolute risk reduction (ARR) of all-cause death by CABG with internal thoracic artery graft and consequent number needed to treat (NNT) are shown.

**Central Message**

The magnitude of the survival benefit of CABG with internal thoracic artery graft increases with time over decades.

See Commentaries on pages 112 and 114.

## Magnitude of Reduction in All-Cause Mortality With Treatment of Coronary Artery Disease: Absolute Risk Reduction and Consequent Number Needed to Treat

Treatments for coronary artery disease (angina and myocardial infarction) have emerged in the following order: nitroglycerin in the late 1870s, coronary artery bypass grafting (CABG) in the early 1960s, percutaneous coronary intervention (PCI) in the late 1970s, and statins in the late 1980s. CABG was expected to reduce mortality and prevent myocardial infarction from the beginning, and several randomized trials and observational studies that investigated the outcomes of CABG versus drug treatment were already planned and conducted in the first decade. However, at that time, the operative mortality was higher in the CABG group, and the incidence of myocardial infarction was reported to be similar or even higher in the CABG group than in the drug group. The effectiveness of CABG surgery in preventing all-cause mortality was first reported in a meta-analysis by Yusuf and colleagues^1^ in 1994, approximately 30 years after the CABG was first performed. If a treatment is judged to be effective on the basis of the results of multiple randomized trials or meta-analyses, this level A evidence leads to a class I recommendation for the treatment in the guidelines. The treatment then becomes the first choice in clinical practice. However, as medical management and PCI for coronary disease have evolved over the years, the decision to actually perform an invasive procedure such as a CABG, should be not only on the basis of the “presence or absence of treatment effect,” but also on the “magnitude of treatment effect” being worthy of surgery. For example, a 20% reduction in the relative risk of death might sound quite impressive, but its effect on the patients and our surgeons' practice might nevertheless be minimal. This notion is illustrated using a concept called “number needed to treat” (NNT); the number of patients who must undergo CABG during a specific time to prevent 1 adverse outcome. It is the reverse of the absolute risk reduction (ARR). The presence or absence of a treatment effect or superiority can be assessed to some extent in observational studies, but the magnitude of the treatment effect (expressed as NNT) for a given group of patients can only be assessed in randomized trials. Therefore, for cardiac surgeons who are in charge of life and death, assessing the magnitude of the reduction in all-cause mortality (ie, NNT of all-cause mortality), is extremely important when deciding whether or not to perform surgery.

In 1977, Gruentzig was the first to successfully perform PCI. In the 1980s, randomized trials and meta-analysis showed that PCI for acute myocardial infarction reduced short-term mortality by 9% to 7%, an ARR of 2% (9% − 7% = 2%).^2^, ^3^, ^4^ The inverse of this ARR (that is, 1 divided by the ARR) is equal to the number of such patients cardiologists would have to treat to prevent 1 death—the NNT. In this case, the cardiologist would have to treat 50 such patients to save a single life (1/0.02 = 50). Statins were approved by the US Food and Drug Administration in 1987, and as early as 1994, a randomized trial (4S) reported that statins were effective in preventing myocardial infarction and improving life expectancy.^5^ In 2005, a meta-analysis that combined 14 randomized trials since the 4S trial (totaling 90,056 patients) reported that statin therapy for secondary prevention improved the 4.7-year survival rate by 1.2% corresponding to a 4.7-year NTT of 83.^6^ On the basis of the results of these randomized trials and a meta-analysis, global guidelines classify PCI for acute myocardial infarction and statins for hypercholesterolemia recommended as class I with level of evidence A.

## CABG Has a Preventive Effect on All-Cause Mortality (Level of Evidence A)

**Figure 1:**
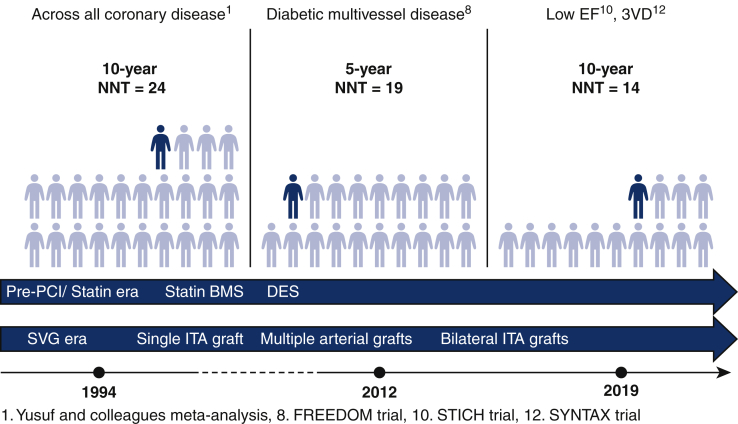
Number needed to treat (*NNT*) of coronary artery bypass grafting (CABG) to prevent 1 death across landmark trials in patients with coronary artery disease.^1^^,^^7^^,^^9^^,^^11^ The coronary artery diseases for which CABG has a significant prognostic effect are diabetic multivessel disease, low ejection fraction (*EF*), and 3-vessel disease (*3VD*). Despite advances in drug therapies such as statins and percutaneous coronary intervention (*PCI*) devices (bare-metal stents [*BMS*], drug-eluting stents [*DES*]), the magnitude of the survival benefit of CABG using the internal thoracic artery (*ITA*) compared with the era of SVG is greater at 10 years. This might be in part because of the more recent use of multiple arterial grafting and bilateral ITA grafting in CABG. *SVG*, Saphenous vein graft; *FREEDOM*, Future Revascularization Evaluation in Patients with Diabetes Mellitus: Optimal Management of Multivessel Disease; *STICH*, Surgical Treatment for Ischemic Heart Failure; *SYNTAX*, SYNergy between percutaneous coronary intervention with TAXus and cardiac surgery.

**Figure 2:**
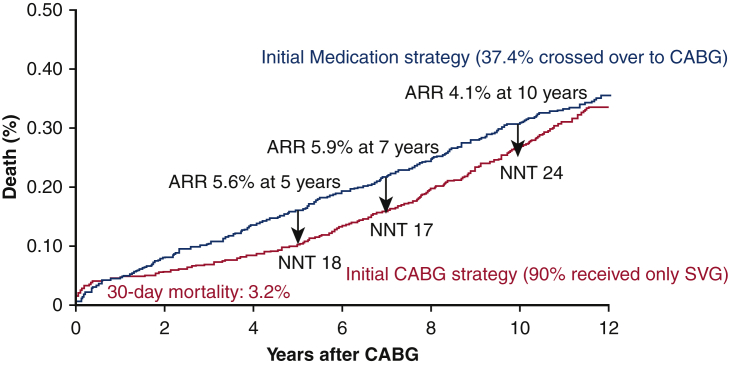
Absolute risk reduction (*ARR*) of all-cause death for saphenous vein coronary artery bypass and consequent number needed to treat (*NNT*) in the meta-analysis from Yusuf and colleagues.^1^ In the era of SVG, coronary artery bypass grafting (*CABG*) reduced all-cause mortality by 5.6% at 5 years, 5.9% at 7 years, and 4.1% at 10 years. The magnitude of the survival benefit was greatest at approximately 7 years postoperatively, then decreased, and by 12 years, the effect had disappeared. *SVG*, Saphenous vein graft.

The first meta-analysis was by Yusuf and colleagues^1^ in 1994, who reported that the strategy of initial CABG surgery is effective in preventing all-cause mortality (Table 1 and Figure 1). In the report by Yusuf and colleagues the 30-day mortality rate in the CABG group was relatively high at 3.2%. Nonetheless, CABG reduced all-cause mortality by 5.6% at 5 years, 5.9% at 7 years, and 4.1% at 10 years (Figure 2). The magnitude of this treatment effect was greatest at approximately 7 years postoperatively, then decreased, and by 12 years, the effect had disappeared. The level A evidence from the meta-analysis by Yusuf and colleagues has long been the fundamental reason for cardiac surgeons to perform CABG. To date, the European and Japanese guidelines have given class I recommendations for CABG in all stable coronary artery disease patients with left main trunk (LMT) lesions or/and proximal left anterior descending lesions. However, there are now critics and detractors of the all-cause mortality benefit of CABG as reported by Yusuf and colleagues. The criticism comes mainly from the cardiologist community. They argue that this report is a synthesis of randomized trials conducted at a time when statins and PCI were not yet actively performed, and that CABG might no longer be effective in preventing all-cause mortality, or even if it is, it might be less effective. The counter argument to this criticism comes from the cardiac surgeon community: Yusuf and colleagues reported that 90% of CABGs used only vein grafts, so the effect was small after 7 years and disappeared after 12 years. However, modern CABG uses at least 1 internal thoracic artery (ITA) graft in principle, so the effect might last much longer.

**Table 1:** ARR and NNT of CABG to prevent 1 death across landmark trials in patients with coronary artery disease

	Study	Coronary artery disease	Patient n	All-cause mortality			ARR, %	NNT	*P* value
				Non-initial CABG strategy, %	Initial CABG strategy, %	Years after CABG			
SVG and pre-statin era	Yusuf and colleagues^1^ meta-analysis	3VD 51%, 2VD 32%, 1VD 10%, LMD 7%, DM 10%, low EF 7%	2649	15.8	10.2	5	5.6	18	<.0001
				21.7	15.8	7	5.9	17	<.001
				30.5	26.4	10	4.1	24	.03
ITA and statin era	FREEDOM^7^	Diabetic multivessel disease	1900	16.3	10.9	5	5.4	19	.049
	STICH^8^^,^^9^	Low EF (≤35%)	1212	41	36	5	5.0	20	.12
				66.1	58.9	10	7.2	14	.02
	SYNTAX^10^^,^^11^	3VD	1095	14.6	9.2	5	5.4	19	.006
				28.0	21.0	10	7.0	14	<.05
	EXCEL^12^^,^^13^	LMD (SYNTAX Score <33)	1905	8.2	5.9	3	2.3	43	.11
				13.0	9.9	5	3.1	32	<.05

*ARR*, Absolute risk reduction; *NNT*, number needed to treat; *CABG*, coronary artery bypass grafting; *SVG*, saphenous vein graft; *VD*, vessel disease; *LMD*, left main disease; *DM*, diabetes mellitus; *EF*, ejection fraction; *ITA*, Intenal Thoracic Artery; *FREEDOM*, Future Revascularization Evaluation in Patients with Diabetes Mellitus: Optimal Management of Multivessel Disease; *STICH*, Surgical Treatment for Ischemic Heart Failure; *SYNTAX*, SYNergy between percutaneous coronary intervention with TAXus and cardiac surgery; *EXCEL*, Evaluation of XIENCE versus Coronary Artery Bypass Surgery for Effectiveness of Left Main Revascularization trial.

Yusuf and colleagues also noted that the groups with the greatest reduction in all-cause mortality with CABG were those with LMT disease, low cardiac function, and 3-vessel disease. The Bypass Angioplasty Revascularization Investigation (BARI) trial, published in 1996, reported that those with diabetic multivessel disease was a group that showed a significant benefit in preventing all-cause mortality with CABG.^14^ Since the report from Yusuf and colleagues, the development of aggressive medical therapy and PCI with stents has resulted in the following groups of coronary lesions being prioritized for CABG in clinical practice: LMT disease, 3-vessel disease, and diabetic multivessel disease. Furthermore, with the advent of PCI using drug-eluting stents, randomized trials for these lesion groups were planned in the 2000s, and the results of these trials have been reported since the 2010s.

The Future Revascularization Evaluation in Patients with Diabetes Mellitus: Optimal Management of Multivessel Disease (FREEDOM) trial was reported in 2021, which randomized patients who had diabetes and multivessel coronary disease to PCI and CABG.^7^ In the CABG group 82.6% of the patients were receiving statins and ITA was used in 94.4% of the patients. The 5-year mortality rate was 10.9% in the CABG group and 16.3% in the PCI group (*P* = .049); CABG reduced all-cause mortality by 5.4% over 5 years, corresponding to a 5-year NTT of 18.5 (= 100/5.4).

The Surgical Treatment for Ischemic Heart Failure (STICH) trial was a randomized trial of patients with low cardiac function with an ejection fraction of <35%.^8^^,^^9^ It compared a medical therapy group (602 patients) with a CABG group (610 patients). Five-year follow-up was reported in 2011, which showed no significant difference in all-cause mortality. However, the 10-year results reported in 2016 showed that the all-cause mortality was 66.1% in the drug group versus 58.9% in the CABG group (*P* = .02). CABG reduced all-cause mortality by 7.2% over 10 years, corresponding to a 10-year NNT of 13.9 (= 100/7.2).

The SYNergy between percutaneous coronary intervention with TAXus and cardiac surgery (SYNTAX) trial reported in 2014 showed that in patients with 3-vessel disease, 5-year all-cause mortality was 14.2% for PCI with drug-eluting stents and 9.2% for CABG (*P* < .05); CABG reduced all-cause mortality by 5.0% over 5 years, corresponding to a 5-year NNT of 20 (= 100/5.0).^10^ ITA grafting was done in 97% of patients in the CABG group. In 2019, the 10-year results of the SYNTAX trial were reported: in patients with 3-vessel disease, 10-year all-cause mortality was 28% for PCI and 21% for CABG; CABG resulted in a 7% reduction in all-cause mortality over 10 years, corresponding to a 10-year NNT of 14.2 (= 100/7).^11^ In the Evaluation of XIENCE versus Coronary Artery Bypass Surgery for Effectiveness of Left Main Revascularization (EXCEL) trial of patients with LMT disease with a SYNTAX score of 32 or less, the reduction in all-cause mortality with CABG was 2.3% at 3 years (8.2% PCI vs 5.9% CABG) and 3.1% at 5 years (13.0% PCI vs 9.9% CABG; *P* < .05).^12^^,^^13^

On the basis of the results of the 4 randomized trials mentioned herein, CABG reduced all-cause mortality in patients with LMT disease, low cardiac function, 3-vessel disease, and diabetic multivessel disease. Therefore, we can conclude that CABG still has a preventive effect on all-cause mortality (level of evidence A), even in the current era of aggressive drug therapy and PCI with drug-eluting stents. More importantly, the magnitude of the reduction in all-cause mortality with CABG using saphenous vein graft (SVG) alone was smaller at 10 years than at 5 years, but the magnitude of the effect with CABG using the ITA graft was larger at 10 years than at 5 years.

## CABG Has a Preventive Effect on Myocardial Infarction (Level of Evidence A)

Although PCI was started in 1977, the scientific terms, “protected” or “unprotected” LMT lesions was introduced as early as the mid-1980s, when the efficacy of CABG in preventing myocardial infarction had not yet been proven by randomized trials. Protected is defined as the presence of a patent bypass graft to the left coronary circulation. In 2008, Daemen and colleagues^15^ reported a meta-analysis of randomized trials that compared PCI with bare-metal stents and CABG, and reported that the incidence of myocardial infarction was similar in both groups. The first randomized trial to show the protective effect of CABG was the BARI 2D trial, which was reported in 2009.^16^ The fact that the term, “unprotected” lesion was coined more than 20 years before the BARI 2D trial reported the protective effect of CABG, and that it is still used today, strongly suggests that the protective effect of patent bypass grafts is clear to the cardiologist community. In the BARI 2D trial aggressive medical therapy alone was compared with aggressive medical therapy plus CABG in diabetic patients who were eligible for CABG. The characteristics of this study were: (1) the statin administration rate was 95% and low-density lipoprotein cholesterol was reduced to 80 mg/dL in both groups, and (2) 39.7% of patients in the aggressive medical therapy group required coronary revascularization later during the follow-up period. Even with statin use at 95% and low-density lipoprotein cholesterol reduction to 80 mg/dL, the strategy of initial CABG surgery reduced the 5-year incidence of myocardial infarction by an additional 7.6% (17.6%-10.0%; *P* = .002), corresponding to a 5-year NNT of 13.1 (= 100/7.6). The subsequent Coronary Artery Revascularization in Diabetes (CARDia),^17^ FREEDOM,^7^ and SYNTAX^18^ trials also reported lower rates of myocardial infarction in the CABG group. The Nordic-Baltic-British left main revascularisation study (NOBLE)^19^ and EXCEL^13^ trials of unprotected LMT disease also reported low rates of myocardial infarction during follow-up, except for perioperative myocardial infarction in the CABG group. Therefore, here again, we can conclude that CABG has a preventive effect on myocardial infarction (level of evidence A), even in the current era of aggressive medical therapy and PCI with drug-eluting stents.

## In Principle, CABG Should Be Performed First When Indicated

In patients with stable coronary artery disease, the results of randomized trials and meta-analysis comparing Percutaneous Transluminal Coronary Angioplasty (so-called POBA: plain old balloon angioplasty) and PCI with bare-metal stents, as well as bare-metal stents and drug-eluting stents, reported that PCI has improved restenosis rates with advances in devices, but not all-cause mortality and myocardial infarction.^20^^,^^21^ The results of the BARI IID^16^ and Clinical Outcomes Utilizing Revascularization and Aggressive Drug Evaluation (COURAGE)^22^ trials also showed that PCI did not improve all-cause mortality or myocardial infarction rates compared with optimal medical therapy. These randomized trials were intention-to-treat analyses, and patients initially assigned to the medical therapy-alone arm also underwent PCI during the follow-up period if deemed necessary. Therefore, we cannot conclude that PCI is ineffective, but we can say that the strategy of PCI first was not superior to the strategy of drug-alone first (drug-alone first, then PCI if needed during follow-up). In contrast to PCI, the results of randomized trials on CABG, such as the meta-analysis by Yusuf and colleagues,^6^ showed that 37.4% of patients in the medical therapy-alone group underwent CABG later during follow-up (ie, cross-over), yet the group initially assigned to CABG had better survival. In the BARI 2D trial, which was the first to provide evidence of the preventive effect of CABG on myocardial infarction, 39.7% of the patients in the active drug treatment group underwent coronary revascularization during follow-up.^16^ The STICH trial also showed that 17% of patients in the treatment group underwent CABG within 5 years and 20% within 10 years.^8^^,^^9^ The treatment effect of CABG is prophylactic, so of course, if surgery is indicated, it must be done as soon as possible or the effect will be small.

## Good Patency of ITA Grafts is Constant and Unchanged Over 20 Years (Level of Evidence B)

In 1996, Cameron and colleagues^23^ compared the long-term survival of the CABG group with ITA grafts (749 patients) and the SVG-alone group (4888 patients) in an observational study and reported that the group with ITA grafts had consistently better survival for 15 years after surgery. The dissociation of the life curves in the 2 groups became more pronounced after the eighth year, which is considered to be the life span of the SVG, and extended to the 15th year. In 2017, Raza and colleagues^24^ reported graft patency rates for 57,961 patients who underwent isolated CABG at the Cleveland Clinic; SVG patency rates decreased over time (70% at 5 years, 57% at 10 years, and 41% at 20 years), whereas ITA graft patency rates of 95% were constant and unchanged for 20 years postoperatively. In 2018, Head and colleagues^25^ reported the results of a pooled analysis of data from 11 randomized trials (11,518 patients). They reported a 2.0% reduction in 5-year all-cause mortality with CABG (ITA grafting rate, 96.2%) across all coronary artery disease (2-vessel disease 29.0%, 3-vessel disease 43.2%, LMT disease 38.0%), corresponding to a 5-year NNT of 50 (= 100/2.0). This is level A evidence. The cardiologist community considers this 5-year NNT of 50 to be small and prioritize CABG over PCI only for coronary lesions in which CABG reduces 5-year all-cause mortality by 5% or more (eg, 3-vessel disease, diabetic multivessel disease), and the cardiac surgeon community also does not seem to recognize the importance of this 2% reduction. However, unlike the era reported by Yusuf and colleagues when CABG was performed using SVG alone,^1^ ITA is commonly used in modern CABG, so the 2.0% reduction in all-cause mortality at 5 years^25^ is considered significant. The magnitude of the expected reduction of all-cause mortality with ITA grafting is shown in the Central Image, and although 2% at 5 years is small, it becomes 7% at 15 years and 10% at 20 years.

## Future Directions

**Figure 3:**
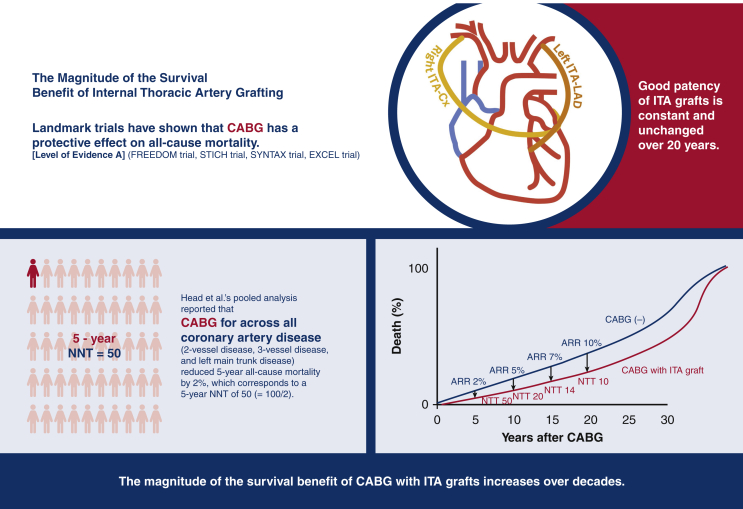
Summary of the magnitude of the survival benefit of internal thoracic artery grafting. *CABG*, Coronary artery bypass grafting; *ITA*, internal thoracic artery; *FREEDOM*, Future Revascularization Evaluation in Patients with Diabetes Mellitus: Optimal Management of Multivessel Disease; *STICH*, Surgical Treatment for Ischemic Heart Failure; *SYNTAX*, SYNergy between percutaneous coronary intervention with TAXus and cardiac surgery; *EXCEL*, Evaluation of XIENCE versus Coronary Artery Bypass Surgery for Effectiveness of Left Main Revascularization trial; *ARR*, absolute risk reduction; *NTT*, Number Needed to Treat.

Considering that the magnitude of the survival benefit of CABG with ITA increases over decades, as summarized in Figure 3, my opinion is that the strategy of choosing initial CABG surgery might be appropriate for younger age with longer life expectancy as well as diabetes, 3-vessel disease, and low cardiac function. In the next revision of the guidelines, I would like to recommend that patient age be an important factor in determining the indication for CABG.

### Conflict of Interest Statement

The author reported no conflicts of interest.

The *Journal* policy requires editors and reviewers to disclose conflicts of interest and to decline handling or reviewing manuscripts for which they may have a conflict of interest. The editors and reviewers of this article have no conflicts of interest.
